# PET imaging facilitates antibody screening for synergistic radioimmunotherapy with a ^177^Lu-labeled αPD-L1 antibody

**DOI:** 10.7150/thno.45540

**Published:** 2021-01-01

**Authors:** Jingyun Ren, Mengxin Xu, Junyi Chen, Jie Ding, Peipei Wang, Li Huo, Fang Li, Zhibo Liu

**Affiliations:** 1 Department of Nuclear Medicine, Peking Union Medical College Hospital, Chinese Academy of Medical Science & Peking Union Medical College, Beijing Key Laboratory of Molecular Targeted Diagnosis and Therapy in Nuclear Medicine, Beijing, China.; 2 Radiochemistry and Radiation Chemistry Key Laboratory of Fundamental Science, Beijing National Laboratory for Molecular Sciences, College of Chemistry and Molecular Engineering, Peking University, Beijing, 100871, China.; 3 Peking University-Tsinghua University Center for Life Sciences, Beijing, 100871, China.

**Keywords:** Immune checkpoint blockade (ICB), αPD-L1, Lutetium-177 (^177^Lu), Radioimmunotherapy (RIT), CD8^+^ T cell

## Abstract

**Rationale:** The low response rate of immunotherapy, such as anti-PD-L1/PD-1 and anti-CTLA4, has limited its application to a wider population of cancer patients. One widely accepted view is that inflammation within the tumor microenvironment is low or ineffective for inducing the sufficient infiltration and/or activation of lymphocytes. Here, a highly tumor-selective anti-PD-L1 (αPD-L1) antibody was developed through PET imaging screening, and it was radiolabeled with Lu-177 for PD-L1-targeted radioimmunotherapy (RIT) and radiation-synergized immunotherapy.

**Methods:** A series of αPD-L1 antibodies were radiolabeled with zirconium-89 for PET imaging to screen the most suitable antibodies for RIT. Mice were divided into an immunotherapy group, a RIT group and a radiation-synergized immunotherapy group to evaluate the therapeutic effect. Alterations in the tumor microenvironment after treatment were assessed using flow cytometry and immunofluorescence microscopy.

**Results:** Radiation-synergistic RIT can achieve a significantly better therapeutic effect than immunotherapy or RIT alone. The dosages of the radiopharmaceuticals and αPD-L1 antibodies were reduced, the infiltration of CD4^+^ and CD8^+^ T cells in the tumor microenvironment was increased, and no side effects were observed. This radiation-synergistic RIT strategy successfully showed a strong synergistic effect with αPD-L1 checkpoint blockade therapy, at least in the mouse model.

**Conclusions:** PET imaging of ^89^Zr-labeled antibodies is an effective method for antibody screening. RIT with a ^177^Lu-labeled αPD-L1 antibody could successfully upregulate antitumor immunity in the tumor microenvironment and turn “cold” tumors “hot” for immunotherapy.

## Introduction

Immune checkpoint blockade therapy, such as anti-PD1/PD-L1 and anti-CTLA4, has been highly successful in the clinical treatment of many cancers 1, 2. However, this emerging cancer therapy suffers from a low response rate, limiting its application to a wider population of cancer patients 3, 4. The exact reasons for the general resistance of cancer patients to checkpoint blockade therapy are still unclear and probably vary among different cancers, and preliminary studies have shown that resistance seems to be related to the tumor PD-L1 expression level and immune infiltration status 5-7. Although some studies have found that the treatment response seems to be related to PD-L1 expression 8, it has been reported that patients with PD-L1-negative tumors can also respond to treatment, which may be related to limited tissue sampling or the temporal and spatial heterogeneity of the tumor 9. As one of the mainstream cancer treatment strategies, external radiotherapy could induce DNA damage in rapidly dividing cancer cells, resulting in tumor antigen release and creating a focal inflammatory response 10, which is often considered a key factor that could upregulate the immune response of tumors.

The relationship between radiation and the immune system was proposed 100 years ago 11, yet the effect on bystander cells has been largely ignored for decades. The discovery of immunogenic cell death (ICD) and *in vitro* effects provides formal evidence for the immune effect of radiation 12, 13. The nonpersistent and limited response to checkpoint blockade among patients is a key challenge for cancer immunotherapy 14. The direct and indirect effects of radiotherapy on tumor cells and tumor-related immune cells together determine the extent to which radiotherapy increases tumor immunogenicity and the synergistic effect between radiotherapy and immunotherapy. Sharverdian *et al.* reported that in the cohort of patients enrolled in the KEYNOTE-001 trial (NCT01295827), non-small cell lung cancer (NSCLC) patients who received radiotherapy before pembrolizumab treatment showed better progression-free survival (PFS) and overall survival (OS) than those who did not receive radiotherapy 15. Recently, Liniker *et al.* reported that radiotherapy and αPD-1 antibodies can be safely combined and well tolerated, with no detectable excess toxicity 16.

However, a limitation of external radiotherapy is the limited number of foci lesions that can be targeted, and its practicability is reduced when multiple systemic metastases occur. Therefore, we wondered whether αPD-L1 antibody can be radiolabeled with potent isotopes for internal targeted radioimmunotherapy (RIT). Ideally, the following radiotherapy-induced inflammation could turn “cold” tumors “hot” and then synergize with the checkpoint blockade agent in triggering robust antitumor immunity 17. Though monoclonal antibodies are characterized by a well-defined structure, high binding affinity and long half-life in serum, which make them suitable for targeting tumors 18, they often show high liver accumulation that hampers their application in targeted RIT. An ideal antibody for RIT should have the characteristics of high tumor uptake, long tumor retention and low uptake in the liver, kidney and other major organs. In this paper, we propose an antibody screening strategy based on PET images and performed a systematic PET imaging study of a series of αPD-L1 antibodies, screening the antibody with high tumor-specific uptake and labeling it with the β-emitting radionuclide Lu-177 for RIT and further radiation-synergistic RIT.

## Methods

### Materials

All starting materials were purchased from commercial suppliers (J&K, Sigma-Aldrich, Beijing, China) and were used as received unless otherwise indicated. 11-(4-isothiocyanatophenyl)-3-[6,17-dihydroxy-7,10,18,21-tetraoxo-27-(N-acetylhydroxylamino)-6,11,17, 22-tetraazaheptaeicosine] thiourea (p-SCN-Bn-DFO) and S-2-(4-isothiocyanatobenzyl)-1,4,7,10-tetraazacyclododecane tetraacetic acid (p-SCN-Bn-DOTA) were purchased from Macrocyclics, Inc. (Dallas, TX). An Amicon 50K cut-off ultrafiltration centrifuge was purchased from Millipore Corp., Billerica, MA. The PD-10 column (dead volume 2.5 mL) was purchased from GE Healthcare. Zirconium-89 (3.7 MBq/μL) was purchased from the China Institute of Atomic Energy. Lutetium-177 (40 MBq/μL) was purchased from the ITM Group (Germany). IgG1 isotype control antibody (clone MOPC-21) was purchased from BioLegend.

### Cell lines and experimental animals

Murine colon adenocarcinoma MC38 cells were obtained from the National Infrastructure of Cell Line Resources (Beijing, China). MC38 cells were cultured in RPMI 1640 supplemented with 10% FBS, penicillin (100 IU/mL) and streptomycin sulfate (100 mg/mL) in a humidified atmosphere containing 5% CO_2_ at 37 °C. C57BL/6 male mice (six- to eight-week-old, 18-22 g) were provided by Vital River (Beijing, China).

### Tumor models

We complied with all relevant ethical regulations for animal testing and research. Six- to eight-week-old male C57BL/6 mice were subcutaneously injected in the shoulder with 1 × 10^6^ cells suspended in 100 µL of PBS. The mice underwent imaging and biodistribution studies when the tumors grew to a diameter of ~500 mm^3^, and studies on treatment were initiated when tumor size reached ~100 mm^3^.

### Preparation of ^89^Zr-DFO-αPD-L1/^177^Lu-DOTA-αPD-L1 and radiochemistry

The αPD-L1 antibody was purified using an ultrafiltration centrifuge tube and PBS (pH = 7.4) to remove the L-histidine in the original buffer and stored at 4 °C 19. An aliquot of the antibody stock was then transferred to a 1.5 mL microcentrifuge tube, and the pH of the final solution was adjusted to 8.5-9.0 with sodium tartrate buffer (pH = 9). Finally, 4.0 equivalents of p-SCN-Bn-DOTA or p-SCN-Bn-DFO were added to the solution, which was previously dissolved in DMSO. After incubating for 1 h at 37 °C, the antibody conjugate was purified twice with PBS (pH = 7.4) using an ultrafiltration centrifuge tube. The antibody complex (DOTA-αPD-L1 or DFO-αPD-L1) stock solution was stored at 4 °C.

The conjugated DFO-αPD-L1 solution was transferred to 1.5 mL microcentrifuge tubes and adjusted to the final pH of the resulting solution of 7.0 by adding sodium acetate buffer (pH = 7.0, 100 mM), followed by the addition of an aliquot of ^89^Zr (37 MBq) solution. After incubation at 37 °C for 1 h, the antibody conjugate was purified through PD-10 chromatography. ^89^Zr-DFO-αPD-L1 was eluted with 25 mL fractions of PBS at pH = 7.4 (shown in **Figure S1**). 150 μg of DOTA-αPD-L1 was incubated with 1 mCi of Lu-177 in sodium acetate buffer (pH = 5.5, 200 mM) for 1 h at 37 °C and purified through PD-10 chromatography (shown in **Figure 2A**).

### *In vivo* biodistribution and small animal PET imaging

C57BL/6 mice were subcutaneously injected in the right shoulder with 1 × 10^6^ MC38 cells. When the tumors reached ~500 mm^3^, the mice were intravenously (i.v.) injected with ^89^Zr-DFO-αPD-L1 (3.7 ± 0.1 MBq) and imaged at 2, 12, 24, 48, 72, 96 and 120 h. Approximately 5 min prior to PET/CT image acquisition, the mice were anesthetized through inhalation of a 2% isoflurane/oxygen gas mixture and placed on the scanner bed; a reduced 1.5% isoflurane/oxygen mixture was used to maintain anesthesia during imaging. PET scans were performed on the NanoScan PET-CT scanner (Mediso Medical Solutions, Inc., Hungary). PET data were reconstructed by a Tera-Tomo 3D method. Variance-reduced DW reconstructed PET images were analyzed by Nucline NanoScan software (InterView FUSION, Mediso Medical Solutions, Inc., Hungary) 20, 21.

^89^Zr-DFO-αPD-L1 (0.22 ± 0.01 MBq) was injected intravenously into MC38 tumor-bearing mice. Blood, small intestine, large intestine, pancreas, liver, spleen, kidney, stomach (without content), brain, bone, lung, heart, muscle, fat and tumor samples were collected at 24 and 96 h post injection (n = 4), and then these items were weighed and measured with a gamma counter (FH-421).

### Immunofluorescence microscopy

The representative tissues were fixed in 4% paraformaldehyde and embedded in paraffin. Deparaffinization was carried out with xylene and ethanol gradients, and antigens were recovered with EDTA buffer (pH = 8.0). Then tissues were incubated with BSA for 30 min and added the anti-CD4 mAb (Servicebio, China; GB13064-2) and incubated overnight at 4 ° C. The slides were then washed in PBS buffer for 5 min and then treated with anti-rabbit horseradish peroxidase (HRP)-conjugated secondary mAb (Servicebio, China; GB23303) for 50 min at room temperature (RT). The slides were then washed in PBS buffer for 5 min, added CY3 (Servicebio, China) and incubate for 10 min, transferred into EDTA buffer and heat-treated using a microwave, then cooled in the same solution to RT. The same process was repeated for the following mAbs and fluorescent dyes, in order: anti-CD8 mAb (Servicebio, China; GB13429)/HRP-conjugated secondary mAb (Servicebio, China; GB22303)/FITC (Servicebio, China). Each slide was then treated with two drops of DAPI (Servicebio, China; G1012), washed in distilled water. A coverslip was applied to each slide in the end. All images were acquired by a laser scan confocal microscope (Nikon Eclipse C1, Japan).

### Flow cytometry analysis

Mouse tumor tissue was obtained, and necrotic tissues and fat were removed.

The tumor tissue was cut into small pieces (approximately 2 mm) and treated with 1 mg/mL collagenase I (Gibco, USA) for 1 h and then ground with the rubber end of a syringe. Cells were filtered through nylon mesh filters and washed with PBS. PBS containing 5% FBS was added for resuspension, and the cell concentration was adjusted to 10^7^ cells/mL for use. The cells were further stained with the following fluorochrome-conjugated antibodies: FITC anti-mouse CD3 (100204), PE anti-mouse CD8a (100708), APC anti-mouse CD4 (100412), PerCP-Cy5.5 anti-mouse CD45 (103132), APC anti-mouse PD-L1 (124312), FITC anti-mouse CD4 (11-0041-81), APC anti-mouse CD25 (17-0251-82), and PE anti-mouse Foxp3 (12-4771-82) (all from BioLegend).

FACS Diva 6.0 Software (BD LSRII) was used for cell acquisition, and data analysis was carried out using FlowJo software (TreeStar, Ashland, OR). All antibodies were diluted 1:200 for use.

### *In vivo* therapy regimen

MC38 cells (1 × 10^6^) were subcutaneously injected into the right shoulder of six- to eight-week-old C57BL/6 mice. The body weights of these tumor-bearing mice were monitored for systemic radiotherapy-related toxicity. Tumor growth was monitored by measurement with a digital caliper, where tumor volumes were calculated as follows: (width^2^ × length)/2. For the immunotherapy group, the mice were divided into a control group (injected with 10 mg/kg IgG1) and an αPD-L1 treatment group (injected with 5 mg/kg αPD-L1 or 10 mg/kg αPD-L1). For the radiotherapy group, the mice were divided into a control group (injected with 11.1 MBq of ^177^Lu-DOTA-IgG1) and a ^177^Lu-DOTA-αPD-L1 group (injected with 3.7 MBq or 11.1 MBq of ^177^Lu-DOTA-αPD-L1). The mice in the radiation-synergized immunotherapy group were injected with 3.7 MBq of ^177^Lu-DOTA-αPD-L1, followed by injection of 5 mg/kg αPD-L1. The treatment methods corresponding to the different groups are shown in **Table 2**.

Five days after tumor inoculation, the αPD-L1 treatment groups were intraperitoneally (i.p.) injected with the given agent once every 2 days for up to 6 times (**Figure 2B**). Five days after tumor inoculation, the ^177^Lu-DOTA-αPD-L1 treatment groups were intravenously (i.v.) injected with the given agent, which was repeated once 7 days later (**Figure 2C**). For the radiation-synergized immunotherapy group, five days after tumor inoculation, the group was i.v. injected with 3.7 MBq of ^177^Lu-DOTA-αPD-L1, followed by i.p. injection of 5 mg/kg αPD-L1 3 times. The treatment cycle was repeated two times in total (**Figure 3A**). When the tumor volume reached 1000 mm^3^, the tumor-bearing mouse was also regarded as dead.

### Statistical analysis

The data were analyzed by IBM SPSS version 23.0 (IBM, Armonk, NY, USA).

Continuous variables with a normal distribution are presented as the mean and standard deviation [SD]. The comparisons of the tumor growth volume and body weight of the mice in different therapeutic study groups were performed by two-way repeated measures ANOVA. The differences in CD4^+^ and CD8^+^ T cells, PD-L1^+^ neoplastic cells and regulatory T cells between different treatment groups were compared by independent Student's t-test.

For survival analysis, the periods at risk of tumor volume oversize (reaching 1000 mm^3^ or death) were defined in days for each mouse. Each observation was separated by 2 days. The risk of an event that did not end in tumor oversize or death was due to the end of the observation period. Exploratory analyses revealed that the relationship between the type of therapies and rate of tumor oversize was consistent across time. Survival curves of different therapeutic study groups were compared by the log-rank test. Statistical significance was set at *p* < 0.05 (*p < 0.05, **p < 0.01, ***p < 0.001).

## Results

### PET imaging-guided antibody screening in MC38 tumor-bearing mice

The ^89^Zr-DFO-Y001, ^89^Zr-DFO-Y002 and ^89^Zr-DFO-Y003 were radiolabeled at an average radiochemical yield of 90.2 ± 2.3%, 89.3 ± 3.5% and 88.2 ± 3.0%, respectively, with greater than 95% for radiochemical purity.

**Figure 1:**
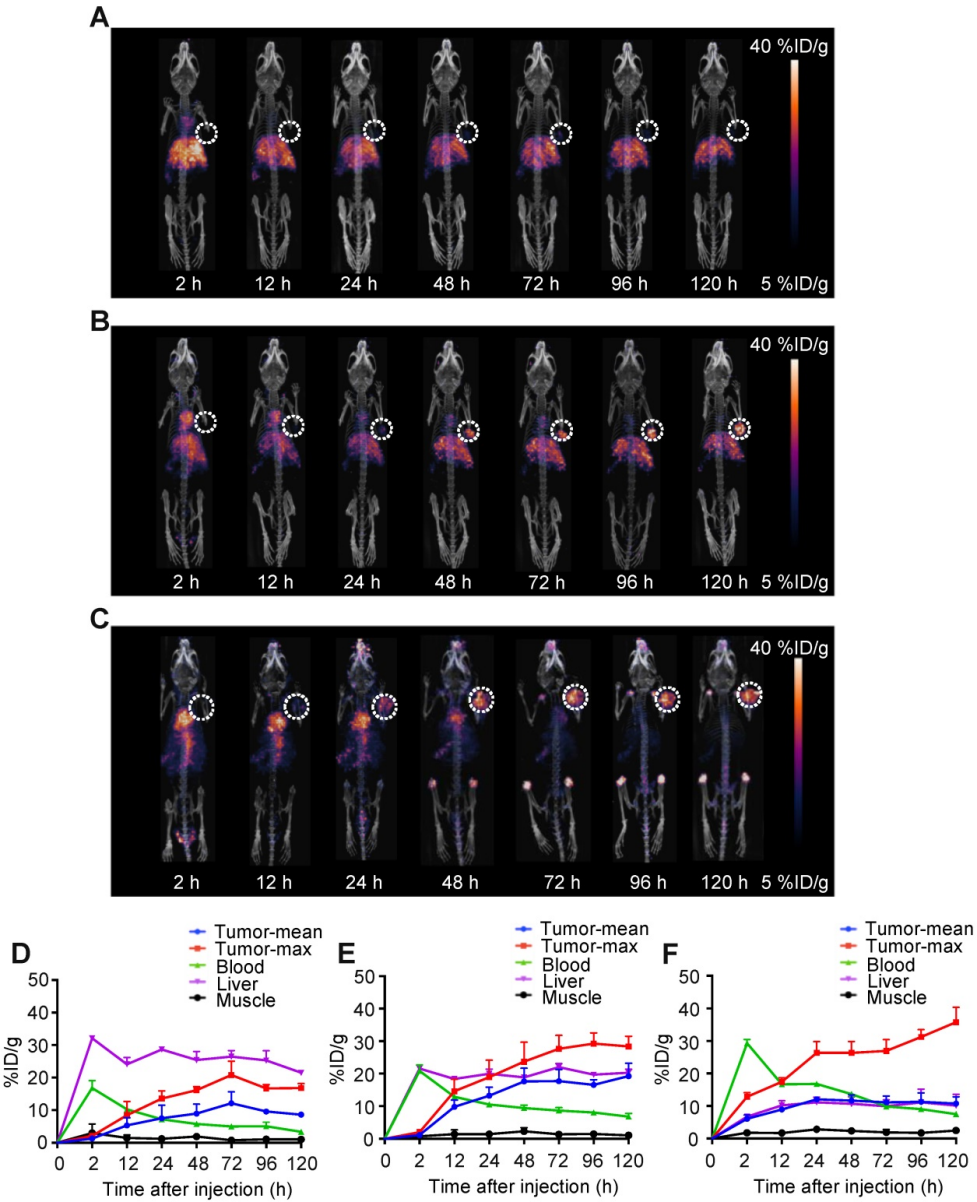
** PET-CT imaging of ^89^Zr-labeled αPD-L1 antibodies identifies Y003 for subsequent RIT because of its remarkable tumor selectivity. A-C.** Representative PET imaging of ^89^Zr-DFO-Y001 (A), ^89^Zr-DFO-Y002 (B) and ^89^Zr-DFO-Y003 (C) at a series of time points in MC38 tumor-bearing mice. **D-E.** Time-activity curves (TACs) of ^89^Zr-DFO-Y001 (D), ^89^Zr-DFO-Y002 (E) and ^89^Zr-DFO-Y003 (F) in the tumor, blood, liver and muscle (n = 4/group).

The *in vivo* biodistributions of ^89^Zr-DFO-Y001, ^89^Zr-DFO-Y002 and ^89^Zr-DFO-Y003 were investigated in MC38 tumor-bearing mice. Static PET-CT imaging at 2, 12, 24, 48, 72, 96 and 120 h post administration was recorded and is shown in **Figure 1A**-**C**. The pharmacokinetics were further interpreted with time-activity curves, which are presented in **Figure 1D-F**.

According to PET imaging, the tumor uptake of ^89^Zr-DFO-Y001 was low (**Figure 1A**). Mild tumor uptake was observed at 24 h after injection. The tumor uptake increased slightly at 48 and 72 h and then decreased at 96 h. At 120 h postinjection, the tumor uptake became negligible. We reason that the low tumor uptake was due to the rapid clearance of ^89^Zr-DFO-Y001 from circulation. At 2 h postinjection, the blood pool uptake could be seen but was minimal. Meanwhile, the liver uptake of ^89^Zr-DFO-Y001 was high at 2 h postinjection and then gradually increased until 120 h after injection, indicating strong evidence of fast hepatobiliary clearance, which is corroborative with the rapid decline of blood uptake. In summary, the poor tumor uptake and unfavorable pharmacokinetics indicate that Y001 is not an optimal candidate for PD-L1-targeted radiotherapy.

As shown in **Figure 1B**, the PET imaging of ^89^Zr-DFO-Y002 exhibited more favorable tumor accumulation than that of ^89^Zr-DFO-Y001. At 2 h postinjection, notable uptake in the cardiac blood pool and abdominal aorta was observed, indicating a remarkably longer blood circulation than that of ^89^Zr-DFO-Y001. The uptake in the blood pool decreased gradually and was almost negligible at 72 h and later time points. Tumor uptake was perceived at 24 h postinjection and inclined gradually until reaching a peak (29 %ID/g) at 96 h. The liver uptake reached the apex (21.5 %ID/g) at the earliest time point and then slowly declined to 20 %ID/g at 120 h postinjection, which is 50% less than that of ^89^Zr-DFO-Y001. According to PET imaging, the most intensive uptake of ^89^Zr-DFO-Y002 was found in the tumor, showing that Y002 is a prospective candidate for immunotherapy but still not good enough for RIT.

Encouraged by the aforementioned progress, we continued the screening until finding Y003. At 2 h postinjection, we found that most of the radioactivity remained in the blood circulation, and almost no liver uptake was observed (**Figure 1C**). Tumor uptake was visible at 12 h postinjection and then continually increased to 40 %ID/g at 120 h postinjection. Compared to ^89^Zr-DFO-Y001 and ^89^Zr-DFO-Y002, the liver uptake of ^89^Zr-DFO-Y003 was almost negligible in PET images at all time points. It is worth noting that the bone uptake became intense at later time points, which could be from the metabolized ^89^Zr-DFO-Y003 that had been internalized in the tumor cells. In fact, except for the bone uptake, the radioactivity could only be found in tumors at time points later than 72 h. Therefore, we chose Y003 as a highly tumor-selective candidate for Lu-177 labeling and subsequent synergistic RIT.

### Biodistribution studies of ^89^Zr-DFO-Y003

The* ex vivo* biodistribution of ^89^Zr-DFO-Y003 was performed at 24 h and 96 h after the injection of ^89^Zr-DFO-Y003. As summarized in **Table 1**, the tumor uptake at 24 and 96 h after injection was 15.18 ± 3.97 %ID/g and 36.76 ± 5.68 %ID/g, respectively (n = 4). The blood uptake of ^89^Zr-DFO-Y003 was high at early time points (up to 23.30 ± 5.94 %ID/g at 24 h after injection), followed by a gradual decrease, and the uptake at 96 h after injection was 5.79 ± 1.32 %ID/g, which is corroborative with PET scans.

### Characterization and radiochemistry of ^177^Lu-DOTA-Y003

**Figure 2:**
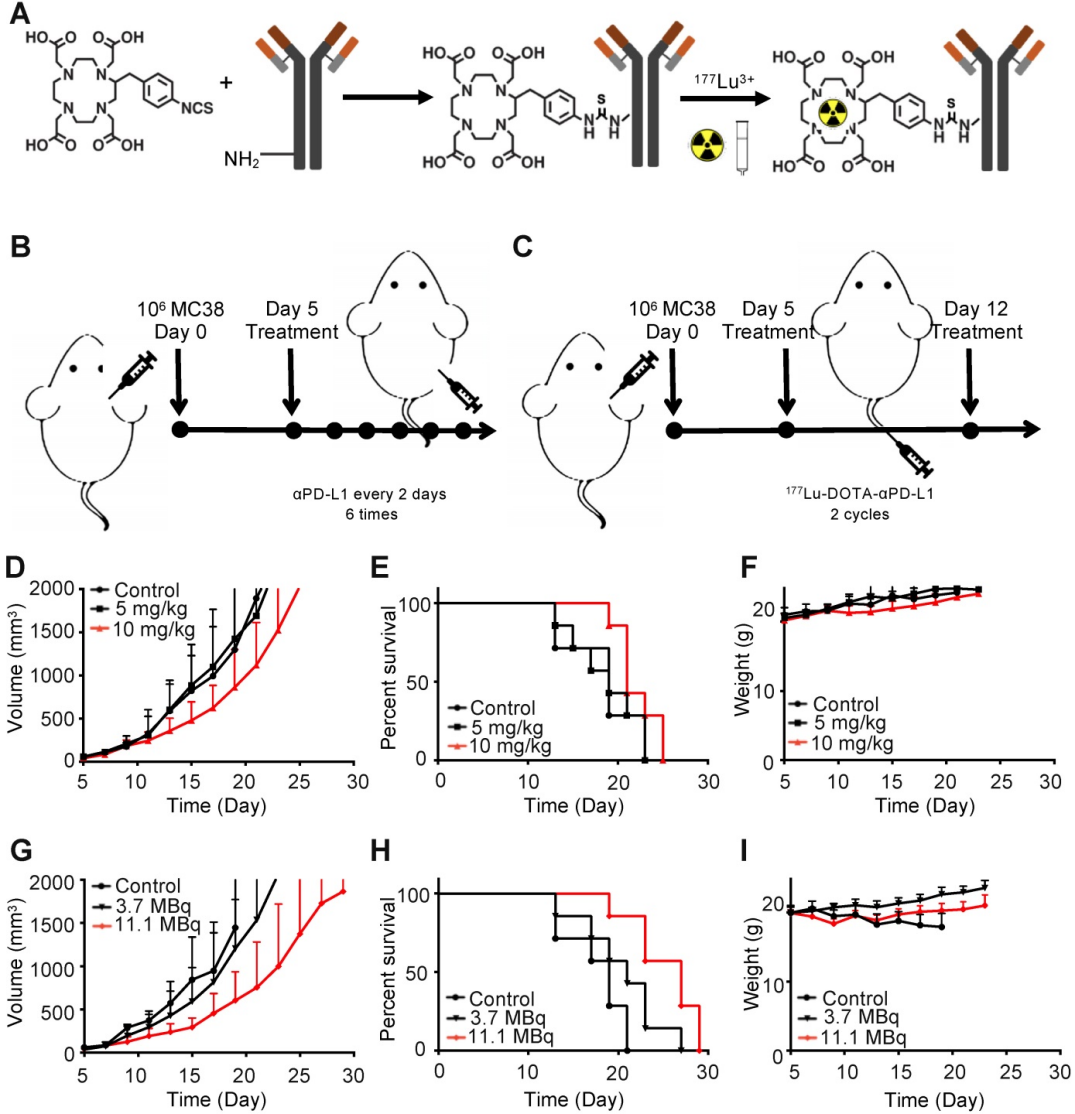
** Antitumor treatment study of ^177^Lu-DOTA-Y003 and Y003 in MC38 tumor-bearing mice. A.** Synthesis and radiolabeling of ^177^Lu-DOTA-Y003. **B-C.** Treatment flow chart of Y003 (B) and ^177^Lu-DOTA-Y003 (C). **D.** Tumor growth curve of Y003 immunotherapy. **E.** Survival curve of mice in the Y003 immunotherapy groups. **F.** Record of body weight in the Y003 immunotherapy groups. **G.** Tumor growth curve of the ^177^Lu-DOTA-Y003 therapy groups. **H.** Survival curve of mice in the ^177^Lu-DOTA-Y003 therapy groups. I. Record of body weight in the ^177^Lu-DOTA-Y003 therapy groups (n = 7/group).

The conjugation scheme and structure of ^177^Lu-DOTA-Y003 is illustrated in **Figure 2A**. The radiochemical yield of ^177^Lu-DOTA-Y003 was 62.7 ± 5.2%, and the purity was > 99% according to instant thin-layer chromatography (iTLC) analysis. The specific activity was calculated to be 250 ± 10 MBq/mg, ^177^Lu-DOTA-Y003 showed good stability *in vitro*, and no decomposition was observed after incubation in PBS for 168 h (**Figure S3**).

### Immunotherapy with Y003 only

The tumor-bearing mice were injected intraperitoneally with 10 mg/kg of IgG1, 5 mg/kg of Y003 or 10 mg/kg of Y003. The treatment was repeated every two days, and each mouse was treated 6 times in total. As shown in **Figure 2D-E**, no difference was observed in either the tumor growth curve or survival curve between the control group and the 5 mg/kg group. The tumor growth of the 10 mg/kg group was slightly slower than that of the former two groups, and the survival period of the mice was also slightly longer than that of the former two groups, indicating that Y003 immunotherapy had a certain therapeutic effect, but it was not good enough to suppress tumor growth by itself. There was no significant difference (*P* = 0.126) in body weight between the treatment group and the control group (**Figure 2F**).

### Radiotherapy with ^177^Lu-DOTA-Y003

Then, we examined whether ^177^Lu-DOTA-Y003 could promote tumor clearance. The tumor-bearing mice were intravenously injected with 11.1 MBq of ^177^Lu-DOTA-IgG1 or 3.7 MBq or 11.1 MBq of ^177^Lu-DOTA-Y003. The treatment was repeated once 7 days after the first treatment. Encouragingly, the low-dose ^177^Lu-DOTA-Y003 treatment group showed slower tumor growth than the control group (**Figure 2G**), though there was no significant difference (*P* > 0.05) in the survival curve (**Figure 2H**). For the high-dose treatment group, the treatment efficacy was remarkable, especially at the early stage of tumor growth. The survival period of the high-dose group was significantly longer than that of the first two groups (P = 0.032), but the weight loss of the mice (**Figure 2I**) was also noteworthy (> 10%, *P* < 0.001), indicating that the high-dose ^177^Lu-DOTA-Y003 treatment could have side effects.

### Synergistic radiotherapy with ^177^Lu-DOTA-Y003+Y003

**Figure 3:**
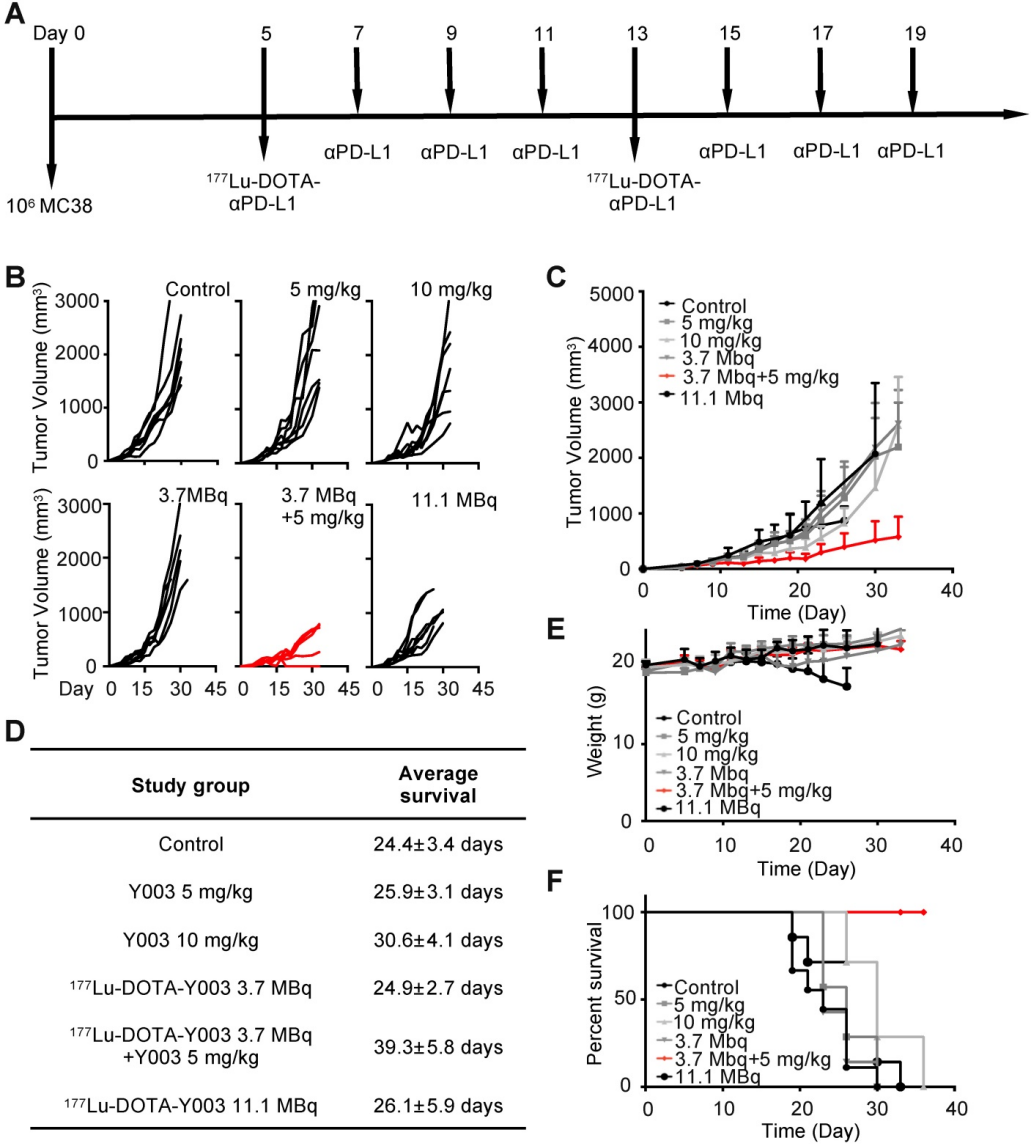
**^177^Lu-DOTA-Y003+Y003 treatment triggers tumor regression in mice. A.** Treatment scheme of ^177^Lu-DOTA-Y003+Y003 in MC38 tumor-bearing mice. **B.** Individual tumor volume at the indicated time point after tumor implantation. **C.** Tumor growth curve of the indicated treatment groups. **D.** Summary of average survival time. **E.** Record of body weight of the indicated treatment groups. **F.** Summary of average survival time (n = 7-9/group).

We reason the poor efficacy of Y003 to the insufficient infiltration and/or inactivation of lymphocytes, and for this reason, checkpoint blockade-resistant tumors are considered to be “cold”. Some colon cancers with high CD8^+^ T cell infiltration and overexpression of regulatory immune checkpoints had a higher response rate to αPD-1 checkpoint blocking immunotherapy 22-24. We wondered whether ^177^Lu-DOTA-Y003-induced inflammation within the tumor microenvironment could “heat” some “cold” tumors and therefore synergize with the checkpoint blockade agent in triggering robust antitumor immunity. To test this idea, the tumor-bearing mice were intravenously injected with ^177^Lu-DOTA-Y003 at day 5 followed by three sequential intraperitoneal injections of Y003 on days 7, 9, and 11 (**Figure 3A**). The treatment cycle was repeated once on day 13 to augment the extent of radiotherapy induction. In control mice treated with 10 mg/kg IgG1, the volume of the MC38 tumors increased by approximately 50-fold in 3 weeks (**Figure 3B**). Strikingly, upon treatment with ^177^Lu-DOTA-Y003+Y003, most of the tumors became reddish in the first few days, and notable tumor shrinkage was observed in some tumors (**Figure 3B**). A pronounced growth delay of approximately 15 days was seen in ^177^Lu-DOTA-Y003+Y003 mice compared with control mice. In contrast, mice treated with ^177^Lu-DOTA-Y003 or Y003 alone behaved similarly to IgG1-treated mice and showed normal and aggressive tumor growth, respectively (**Figure 3B-C**). The survival curve (**Figure 3F**) showed that the survival of the ^177^Lu-DOTA-Y003+Y003 group was significantly prolonged (*P* < 0.001), and the survival rate was 100% at 36 days, while the mice in all the other groups died. The average survival time of the ^177^Lu-DOTA-Y003+Y003 group was also significantly prolonged (*P* < 0.001, **Figure 3D**).

In terms of the side-effect assessment, there were no significant differences (*P* > 0.05) in body weight among the groups treated with 10 mg/kg of IgG1, 5 mg/kg of Y003 only, 10 mg/kg of Y003 only, 3.7 MBq of ^177^Lu-DOTA-Y003 only and radiation-synergized immunotherapy. As observed previously, the body weight of the mice treated with 11.1 MBq of ^177^Lu-DOTA-Y003 decreased significantly (*P* < 0.01, **Figure 3E**), and HE staining also confirmed that the livers of the mice in the high-dose ^177^Lu-DOTA-Y003 group had anatomical structural changes; a large number of hepatocytes showed focal necrosis, nuclear fragmentation or lysis, accompanied by a small number of lymphocytes and neutrophil infiltration (**Figure S5**).

### Radiotherapy with ^177^Lu-DOTA-Y003 successfully tunes the tumor microenvironment

Using flow cytometry, we analyzed PD-L1 neoplastic cells (CD45^-^/PD-L1) and T cells after the administration of combined RIT and radiation-synergized immunotherapy. The tumors in the 3.7 MBq ^177^Lu-DOTA-Y003 group had increased CD8^+^ T cells and CD45^-^/PD-L1 cells compared with those in the control group. Importantly, the addition of αPD-L1 to 3.7 MBq ^177^Lu-DOTA-Y003 resulted in a significant increase in CD8^+^ T cells and CD45^-^/PD-L1 cells compared with the control and 3.7 MBq ^177^Lu-DOTA-Y003 groups. Compared with the control group, the proportion of CD4^+^ T cells in the radiation-synergized immunotherapy group was significantly increased, whereas no significant difference was observed in the 3.7 MBq ^177^Lu-DOTA-Y003 group. There was no significant difference in Treg cells between the control group and the therapeutic groups.

At the end of the treatment, we collected the tumors of mice for immunofluorescence staining to further confirm the effect of the combined treatment on mouse immunity. Anti-CD4 and anti-CD8 fluorescence imaging of the tumor tissue confirmed the remarkably increased T cell infiltration. The therapeutic effect of the ^177^Lu-DOTA-Y003+Y003 group confirmed our hypothesis: the immune response could be enhanced after the injection of ^177^Lu-DOTA-Y003 to achieve the purpose of RIT. In the ^177^Lu-DOTA-Y003+Y003 group, the expression of PD-L1 and CD4^+^ and CD8^+^ T cells increased after ^177^Lu-DOTA-Y003+Y003 treatment. This finding shows that radiotherapy has a certain effect on immunity, and the combination of αPD-L1 therapy can achieve a better immunotherapeutic effect.

## Discussion

As a logical biomarker for predicting the response to αPD-1/PD-L1 immunotherapy, the expression of PD-L1 has been extensively studied in clinical trials since the early development of immunotherapy 5, 25. Currently, the most commonly used predictive biomarker is PD-L1 expression as evaluated in tumor biopsies through immunohistochemical (IHC) staining; however, limitations are present, for example, PD-L1 expression is not always correctly assessed by core needle biopsy and this method cannot reflect the changes in PD-L1 expression over time 26, 27. It is contended that as a probe, the αPD-L1 antibody is more informative, as it provides an image of the entire tumor, both primary and metastatic 28-31. Nevertheless, antibodies could be more limited in their tissue-penetrating capacity and longer circulation time due to their size. Antibodies need to be matched with radiopharmaceuticals with a long half-life (such as Zr-89), and the consequent problem is the radiation dose to healthy tissues and organs. To reduce side effects, we performed a systematic PET imaging study of a series of αPD-L1 antibodies. The PET images and time-activity curves of three representative candidates, denoted as Y001, Y002 and Y003, are presented in this work. Among them, Y003 exhibited remarkably high tumor uptake (up to 40 %ID/g) and particularly rapid clearance from major organs. Thus, it was chosen as the antibody for Lu-177 radiolabeling and subsequent animal studies.

Radioisotope-labeled monoclonal antibodies for RIT are effective cancer treatment strategies because tumor-associated mAbs linked to cytotoxic nuclides can selectively bind tumor antigens and release targeted cytotoxic radiation 17, 32-34. Therefore, these antibodies are an attractive tool to use the immune modulatory function of radiotherapy to transform the immune “cold” environment into a "hot" environment to improve the response rate of immunotherapy. Although the concept of RIT is simple, in practice, it is difficult to achieve substantial clinical success, especially in solid tumors, due to the limited delivery of mAbs in tumors 35, 36. The expression of PD-L1 on tumor cells inactivates CD4^+^ and CD8^+^ T cells through the interaction with PD-1 on their surface 37, and the immune response is stopped 38. Therefore, αPD-1 and αPD-L1 antibodies can reverse the inactivated interaction between tumor cells and T cells, leading to a sufficient immune response and tumor cell killing. In the study of Schaue *et al.* 39, it was observed that tumor-specific T cells increased significantly (*P* < 0.01) in most colorectal cancer (CRC) patients after the completion of radiotherapy. Different doses or fractionations are thought to result in different forms of cell death 40, thus regulating the response of downstream cells. As a result, radiotherapy regimens that induce cell death in the form of immune silencing (i.e., apoptotic cell death) are not expected to synergize with ICB, while the dose that triggers the inflammatory response can be used as an immune modulator to induce the additional effects of radiotherapy and immunotherapy 41. Chen et al. 42 proposed a novel therapeutic regimen that combined αPD-L1 immunotherapy with peptide-based targeted radionuclide therapy and confirmed that this combination therapy can stimulate the infiltration of CD8^+^ T cells in the tumor microenvironment. In our study, the therapeutic effect of Y003 alone and ^177^Lu-DOTA-Y003 RIT on CRC was not satisfactory, but the therapeutic effect of ^177^Lu-DOTA-Y003 radiation-synergized immunotherapy was much better than that of Y003 alone or ^177^Lu-DOTA-Y003 RIT alone, and the combination therapy used a low (50%) radiation dose and antibody dose, with no detectable excess toxicity. Flow cytometry confirmed that after ^177^Lu-DOTA-Y003 injection, CD8^+^ T cell upregulation occurred in the tumor microenvironment, but there was no obvious change in CD4^+^ T cells. More interestingly, after combination therapy, CD4^+^ and CD8^+^ T cells were significantly upregulated in the tumor microenvironment, and no significant increase in Treg cells was observed. Immunofluorescence staining showed that radiation-synergized immunotherapy significantly increased CD4^+^ and CD8^+^ T cells in the tumor microenvironment, which indicated that ^177^Lu-DOTA-Y003 radiation-synergized immunotherapy could achieve double effects, especially at low radiation and antibody doses. It is worth noting that our research not only considers the expression level of PD-L1 in tumor cells, but also considers the state of T cells infiltrating in tumors. As described in a recent review 43, “hot”, “altered” and “cold” tumors were defined based on immune cells in tumor microenvironment, and the tumors are classified according to their immune cells infiltration rather than tumor types, this also explains the superior antitumor effect of the ^177^Lu-DOTA-Y003 radiation-synergized immunotherapy.

It should be noted that our study also has some limitations. First, the study was only tested in a mouse MC38 colon cancer tumor model, which is generally an immune "hot" tumor model that is sensitive to anti-PD-1/PD-L1 therapy. In addition, determining the appropriate dose and timing of RIT is also essential for maximizing the therapeutic efficacy of radiation-synergized immunotherapy, and more studies are needed to find the optimal combination.

## Conclusion

A highly tumor-selective antibody, Y003, was achieved through PET imaging screening as a candidate for Lu-177 labeling and subsequent RIT. We found that the combination of 3.7 MBq of ^177^Lu-DOTA-Y003 and 5 mg/kg Y003 showed remarkably better therapeutic efficacy than 10 mg/kg Y003 immunotherapy or 11.1 MBq ^177^Lu-DOTA-Y003 RIT. The dosages of ^177^Lu-DOTA-Y003 and Y003 were reduced by > 50%, and almost no side effects were observed. ^177^Lu-DOTA-Y003 RIT-induced inflammation within the TME can turn “cold” tumors into “hot” ones. This further indicates that radioisotope (e.g., Lu-177, Ac-225)-labeled antibodies have the potential of improving the efficacy of cancer immunotherapy. Compared to external radiation-induced therapy, this therapeutic combination is also a promising approach for treating metastatic tumors.

## Supplementary Material

Supplementary figures and tables.

## Figures and Tables

**Figure 4:**
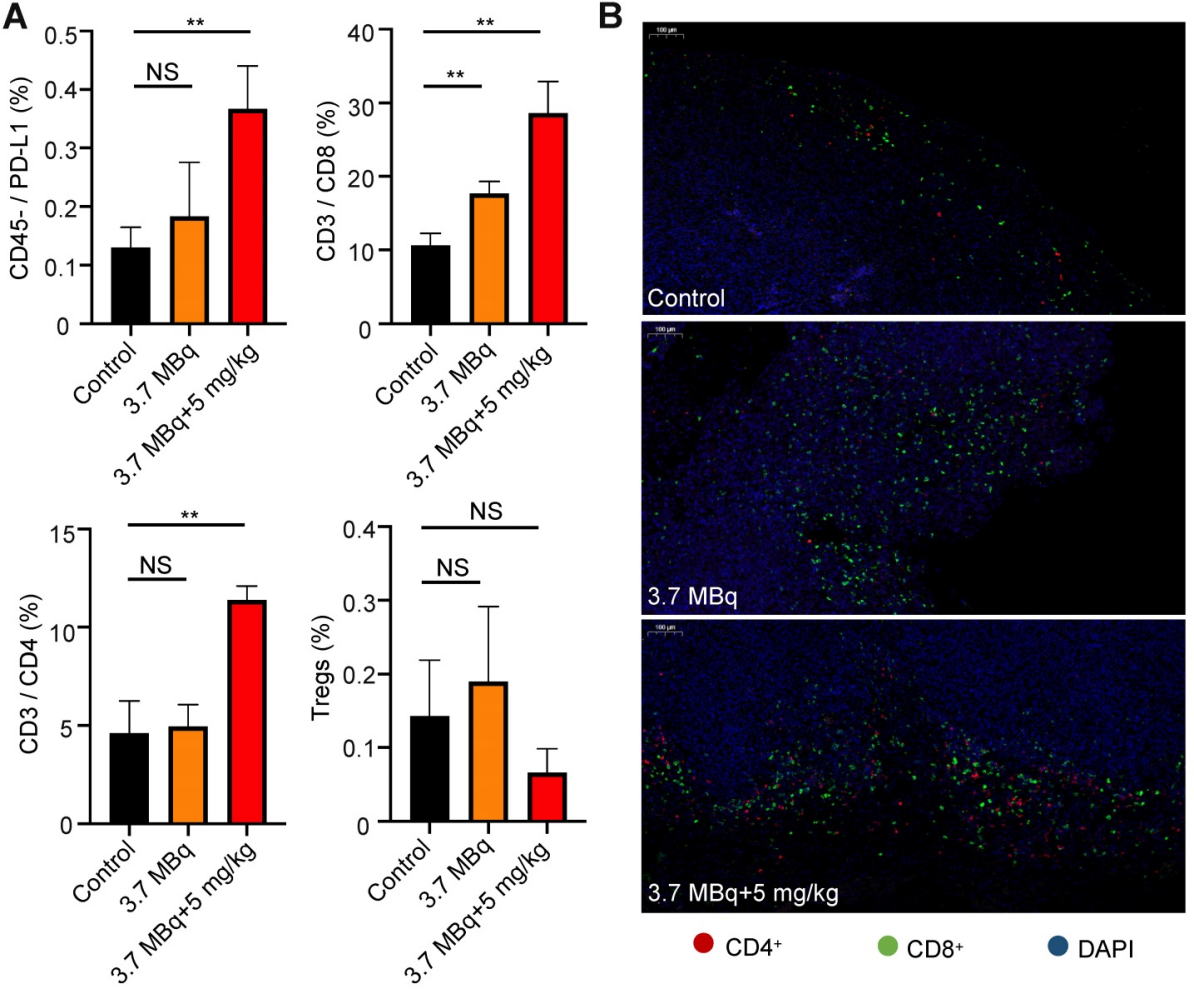
** Treatment with ^177^Lu-DOTA-Y003 increases tumor-infiltrating lymphocytes and upregulates PD-L1 expression in tumors. A.** Percentage of PD-L1 neoplastic cells (CD45^-^/PD-L1), CD8^+^ T cells, CD4^+^ T cells and regulatory T cells in tumors after the indicated treatment. **B.** Representative fluorescent images of the immunofluorescence staining of tumors.

**Table 1:**
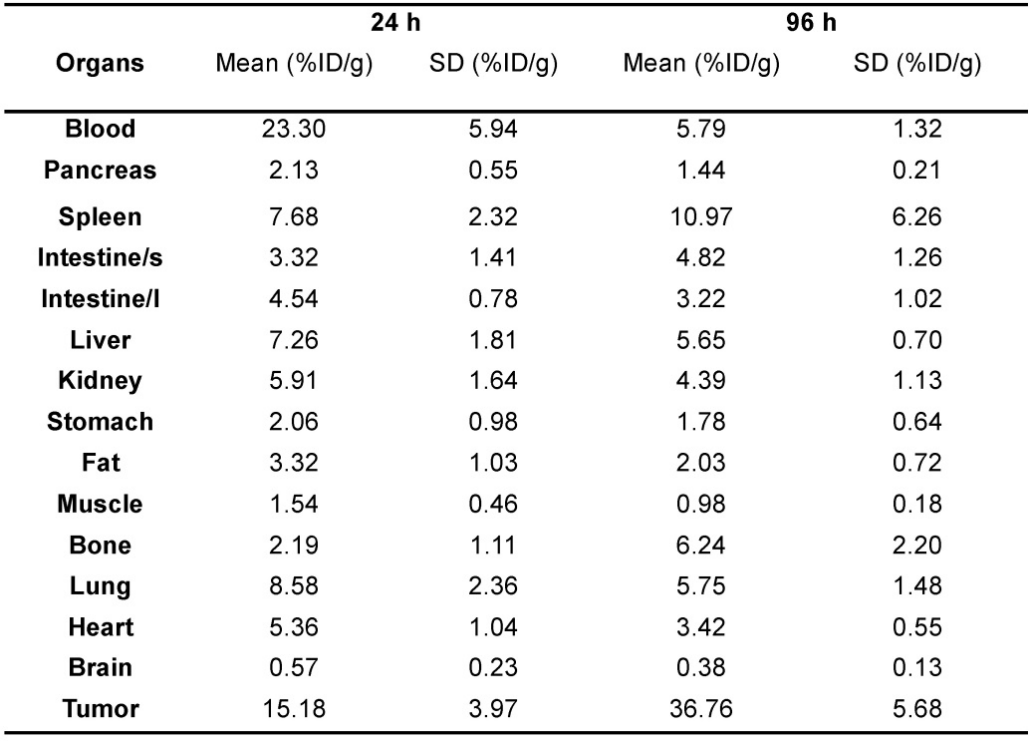
*Ex vivo* biodistribution of ^89^Zr-DFO-Y003 in MC38 tumor-bearing mice at 24 and 96 h postinjection (n = 4/group)

**Table 2:**
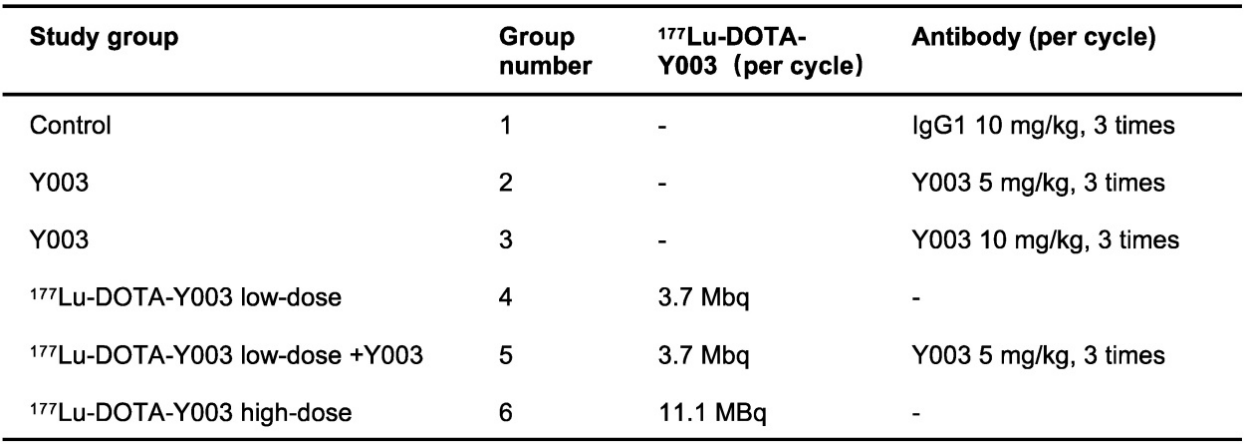
Therapeutic study groups. Mice bearing MC38 colorectal tumors received 2 cycles of injections of the given agent (n = 7-9/group)
