# Supplementary material for: PET imaging facilitates antibody screening for synergistic radioimmunotherapy with a 177Lu-labeled αPD-L1 antibody
Source: Theranostics. 2021 Jan 1;11(1):304–15. doi: 10.7150/thno.45540 (PMC7681088; doi:10.7150/thno.45540)
Supplement: Supplementary file 1 — Supplementary figures and tables. [file thnov11p0304s1.pdf]

Supporting information for

PET Imaging Facilitates Antibody Screening for Synergistic Radioimmunotherapy with a ^{177}Lu-labeled αPD-L1 Antibody

Jingyun Ren[1], Mengxin Xu[2], Junyi Chen[2], Jie Ding[1], Peipei Wang[1], Li Huo[1*], Fang Li[1*], Zhibo Liu[2,3*]

[1]Department of Nuclear Medicine, Peking Union Medical College Hospital, Chinese Academy of Medical Science & Peking Union Medical College, Beijing Key Laboratory of Molecular Targeted Diagnosis and Therapy in Nuclear Medicine, Beijing, China.

[2]Radiochemistry and Radiation Chemistry Key Laboratory of Fundamental Science, Beijing National Laboratory for Molecular Sciences, College of Chemistry and Molecular Engineering, Peking University, Beijing, 100871, China

[3]Peking University-Tsinghua University Center for Life Sciences, Beijing, 100871, China

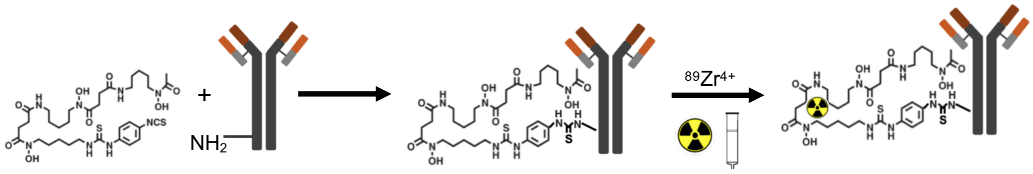

Figure S1: Synthesis and radiolabeling of ^{89}Zr-DFO-αPD-L1.

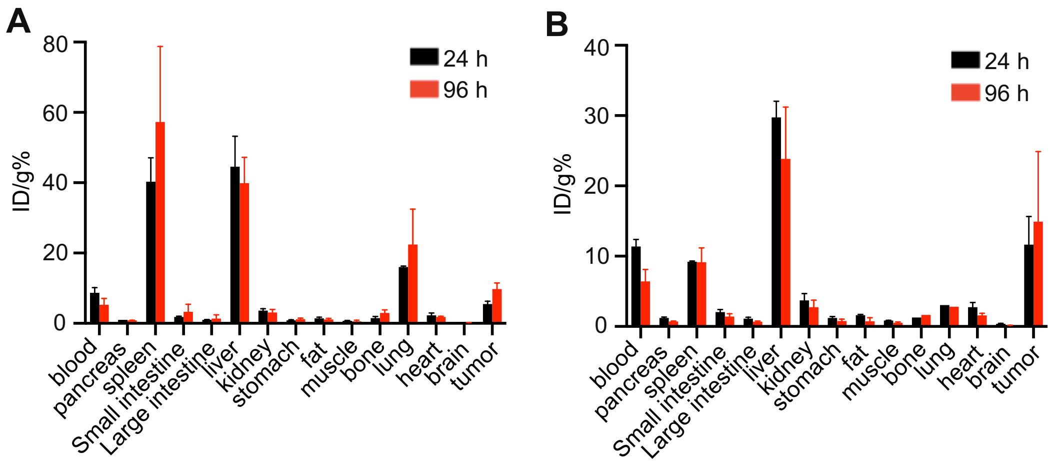

Figure S2: Biodistribution of ^{89}Zr-DFO-Y001 (A) and ^{89}Zr-DFO-Y002 (B) in MC38 tumor bearing mice at 24 and 96 h post injection (n= 4).

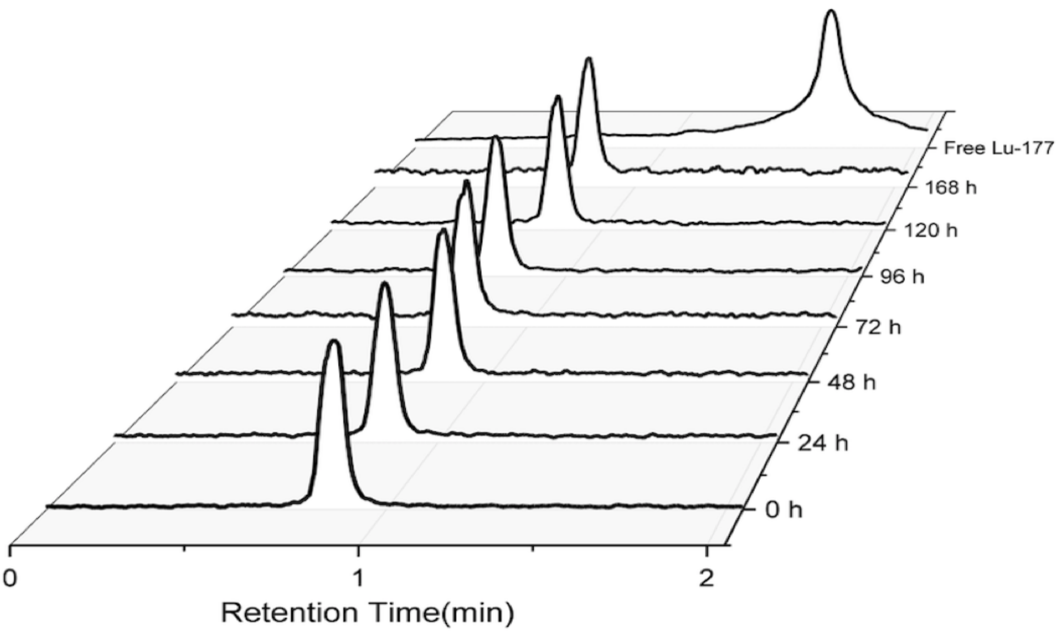

Figure S3: Stability experiment of ^{177}Lu-DOTA-Y003 after incubation in PBS for 168 h.

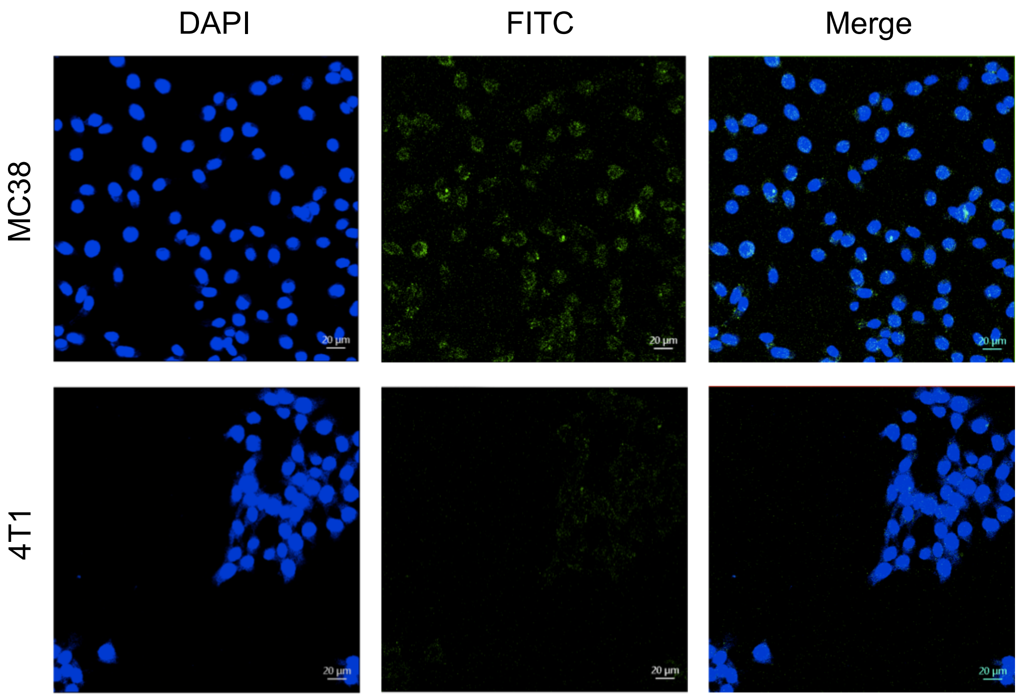

Figure S4: Immunofluorescence for PD-L1 expression in 4T1and MC38 cell lines.

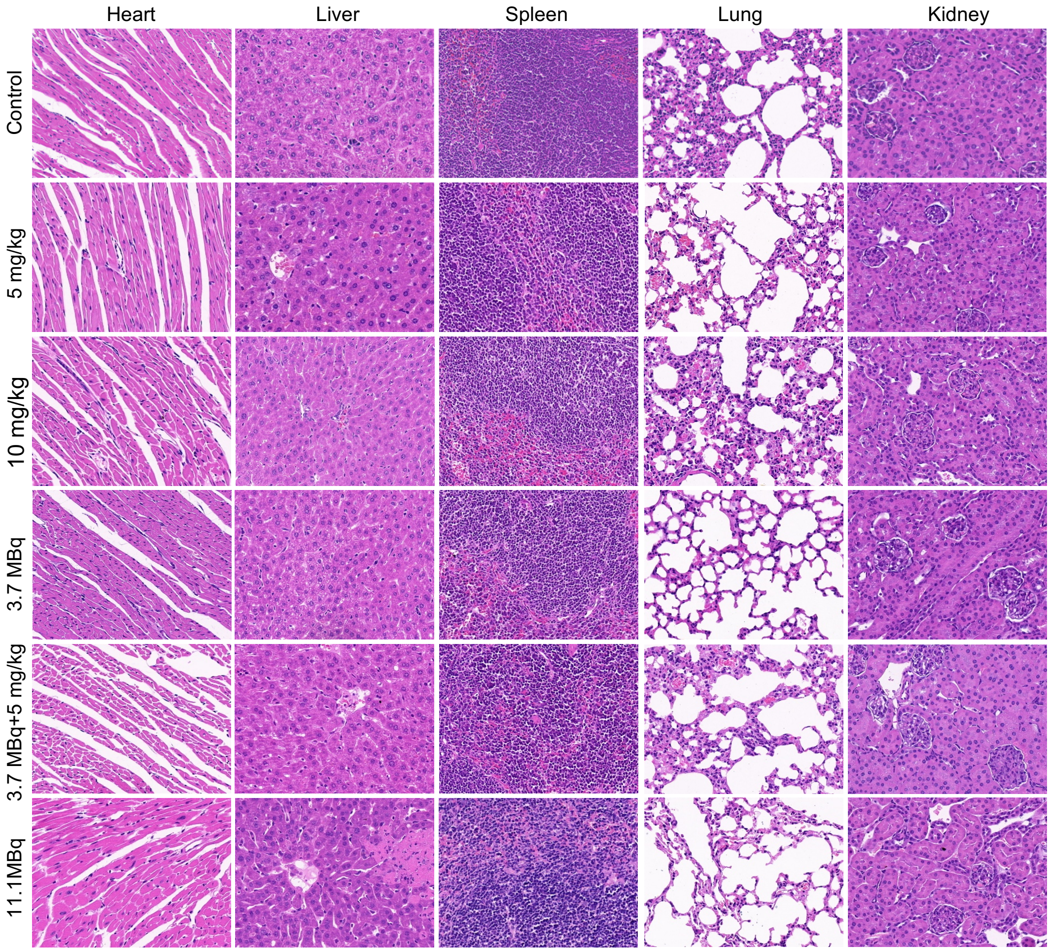

Figure S5. Representative H&E staining of major organ tissues from mice with the indicated treatments (n = 3/group).
